# In Vitro Effectiveness of Soft Contact Lens Solutions Available on the Dutch Market against *Acanthamoeba* Species

**DOI:** 10.3390/pathogens12020214

**Published:** 2023-01-29

**Authors:** Anna C. Randag, Lieke de Kroon, Henny Otten, Cindy Arias Claro-Handgraaf, Barbara Schimmer, Titia Kortbeek, Jeroen van Rooij, Foekje F. Stelma

**Affiliations:** 1 Rotterdam Eye Hospital, Schiedamse Vest 180, 3011 BH Rotterdam, The Netherlands; 2 Rotterdam Ophthalmic Institute, Schiedamse Vest 160, 3011 BH Rotterdam, The Netherlands; 3 Radboud University Medical Center, Postbus 9101, 6500 HB Nijmegen, The Netherlands; 4 Visser Contactlenzen, St. Annastraat 93, 6524 EJ Nijmegen, The Netherlands; 5 National Institute for Public Health and the Environment (RIVM), Postbus 1, 3720 BA Bilthoven, The Netherlands

**Keywords:** *Acanthamoeba castellanii*, *Acanthamoeba polyphaga*, contact lens solutions, Spearman–Karber, XTT colorimetric assay

## Abstract

*Acanthamoeba* keratitis is almost universally associated with contact lens (CL) use. Until today, however, CL solution manufacturing protocols lack testing of anti-amoebic activity. This study investigates the effectiveness of CL solutions available on the Dutch market against trophozoites and cysts of *Acanthamoeba castellanii* and *Acanthamoeba polyphaga*. Sixteen CL solutions were tested: 13 multiple purpose solutions (MPS), 2 hydrogen peroxidase solutions (HPS) and 1 povidone-iodine-based solution (PIS). The Spearman–Karber (SK) log reduction method and an XTT colorimetric assay were used to evaluate the effectiveness at the manufacturer’s minimum recommended disinfection time (MMRDT) and after eight hours. At the MMRDT, one MPS showed an SK mean log reduction (MLR) of >3.0 against *A. castellanii* trophozoites. Two additional MPS and both HPS reached this threshold after eight hours. The SK MLR values for *A. polyphaga* trophozoites were between 1 and 3 at all time points. Using the XTT colorimetric assay, only HPS 1 showed >99.9% reduction (equivalent to 3 log reduction) in metabolic activity of *A. castellanii* trophozoites after eight hours. For *A. polyphaga*, both HPS and PIS showed a metabolic reduction of >99.9% after eight hours. Cysts were resistant against all solutions. We conclude that following the manufacturer’s guidelines, few solutions provide sufficient effectiveness against *Acanthamoeba* trophozoites and none against cysts. The results underline the importance of adequate hygiene when handling CLs.

## 1. Introduction

In 1984, the association between *Acanthamoeba* keratitis (AK) and the use of contact lenses (CLs) was described for the first time [1]. *Acanthamoeba* live ubiquitously in the environment, particularly in soil and water [2]. Contact of CLs with tap water or surface water is one of the well-known risk factors for acquiring *Acanthamoeba* keratitis [3]. Major or minor epithelial traumata, often occurring in contact lens (CL) use, can provide the *Acanthamoeba* an entrance to the corneal stroma and nerves, which in turn can lead to a potentially devastating corneal infection. Over the past decades, the incidence of *Acanthamoeba* keratitis has increased. Carnt et al. describe an almost threefold increase in incidence numbers in South East England, from 18.5 cases per year presenting at Moorfields Eye Hospital in 1997–1999 to 50.3 cases per year in 2011–2016 [4]. In the Netherlands, the annual number of cases increased from 16 in 2009 to 49 in 2015, resulting in an estimated incidence of 1 in 21,000 soft contact lens wearers in 2015 [5].

The first study on the susceptibility of contact lens (CL) disinfection systems against *Acanthamoeba* species appeared in 1986, showing that chemical disinfection systems did not completely kill *Acanthamoeba* trophozoites and cysts [6]. The results of more recent studies on the efficacy of CL disinfection solutions are still worrisome. Kobayashi et al. found that only one out of eight tested multipurpose solutions (MPS) reached 3 log reduction in *Acanthamoeba* trophozoites and none against cysts [7]. Kilvington et al. showed four MPS and two HPS to be effective against *Acanthamoeba* trophozoites, but the efficacy of CL solutions against cysts varied widely [8]. The resistance of cysts against MPS was confirmed by Fedorko et al. [9].

Several authors, including the Food and Drug Administration (FDA), proposed protocols for the evaluation of the effectiveness of CL solutions against *Acanthamoeba* trophozoites and cysts, and a new ISO standard (19045-2) is under development [8,9,10,11,12,13]. Until today, however, official CL solution manufacturing protocols include testing of the antibacterial and antifungal activity but not of anti-amoebic activity [14,15]. The aim of this study was to determine the anti-amoebic effectiveness of widely used soft CL solutions available on the Dutch market.

## 2. Materials and Methods

### 2.1. Selection and Culture of Acanthamoeba Trophozoites and Cysts

Two common laboratory strains of *Acanthamoeba* species, *Acanthamoeba castellanii* (ATCC 50370) and *Acanthamoeba polyphaga* (ATCC 30461), were selected. Trophozoites were grown in peptone yeast extract glucose (PYG) medium (Merck KGaA, Darmstadt, Germany), in T25 tissue culture flasks (Merck KGaA), at 28 °C [16]. Cysts were obtained by the inoculation of trophozoites into Neff’s constant pH encystment medium (NEM, Merck KGaA) and incubation for 7 days at 28 °C, without agitation [17]. The pH was first adjusted to 7.4–7.8 and after 5 h to 9.0 with 1 M NaOH (Merck KGaA) to promote encystment.

### 2.2. Selection of Contact Lens Solutions and Incubation Times

Soft CL solutions widely available on the Dutch market were grouped based on their composition of active and additional ingredients. The list of ingredients was found online or on the CL solution packaging. The selected CL solutions, consisting of 13 multiple purpose solutions (MPS), one povidone iodine solution (PIS) and two hydrogen peroxide solutions (HPS), and their ingredients are presented in the Supplementary Materials Table S1. All CL solution bottles were previously unopened and used for the study within two weeks after opening. MPS 10 was delivered with a CL container with a silver ion coating, which was not used. The PIS and HPS were neutralized directly after opening the bottle by adding the neutralizing discs, conforming to the manufacturer’s instructions. The manufacturer’s minimum recommended disinfection time (MMRDT) is the number of hours of continuous complete immersion of the CL in CL solution as recommended by the manufacturer. Evaluations of effectiveness were performed after four and six hours to cover the MMRDT of most of the CL solutions and after eight hours to simulate overnight disinfection.

### 2.3. Test Implementation and Analyses

A standard solution of 5 × 10^6^ cells/mL was prepared for *A. castellanii* and *A. polyphaga*, containing either trophozoites or cysts. The two methods are described in greater detail by de Kroon et al., and they were performed in triplicate for the two different strains [16].

#### 2.3.1. Spearman–Karber Log Reduction Method

The inhibitory effect of all 16 CL solutions was determined using Spearman–Karber computations [7,18]. Fifty microliters of the standard solution was added to 4.95 mL of each MPS in a 50 mL centrifuge tube (Greiner Bio-One, Thermo Fisher Scientific, Waltham, MA, USA) in order to obtain a first concentration of 5 × 10^4^ cells mL^−1^. To reach the same concentration of 5 × 10^4^ cells/mL, the composition for the PIS and HPS was as follows: 80 µL of standard solution was added to 7.92 mL PIS, 100 µL standard solution to 9.9 mL HPS 1 and 70 µL standard solution to 6.93 mL HPS 2. As a control, 50 µL of standard solution was added to 4.95 mL of one-quarter Ringer’s solution (Thermo Fisher Scientific, Waltham, MA, USA). Incubation of the 50 mL tubes followed at 28 °C for 0 (positive control), 4, 6 or 8 h. At each sampling time, the 50 mL tubes were vortexed, and 0.5 mL was transferred to 4.5 mL Dey-Engley Neutralizing Broth (DENB, Sigma, St Louis, MO, USA) for MPS and to 4.5 mL one-quarter Ringer’s solution for PIS and HPS. Subsequently, four tenfold serial dilutions were made using PYG medium (theoretical concentrations of 5 × 10^3^, 5 × 10^2^, 5 × 10^1^ and 5 × 10^0^ cells mL^−1^). Two hundred microliters of the positive control and each serial dilution was transferred in triplicate to a 96-well plate (Corning® 96 Well plate, Tissue Culture-treated surface, Merck KGaA, Darmstadt, Germany). The plates were incubated at 28 °C over one week for trophozoites and two weeks for cysts, after which the number of wells with *Acanthamoeba* growth, defined as the presence of at least two trophozoites or cysts seen through a Leica DM IRB inverted microscope (Leica, Wetzlar, Germany; 200–400× magnification), was counted. The mean log reduction was calculated for each CL solution using the SK equation [18,19].

#### 2.3.2. XTT Colorimetric Assay and Residual Growth Analysis

A reduction in the number of metabolically active *Acanthamoeba* trophozoites was determined by the XTT colorimetric assay [20,21]. Using one-quarter Ringer’s solution, the standard solution was diluted to 1 × 10^5^ trophozoites/mL, after which four two-fold serial dilutions were made as a reference. Two hundred microliters of each undiluted CL solution was added in triplicate to 48 ‘testing wells’ with a fixed number of 20,000 amoeba/well and to 16 ‘control wells’. The ‘reference wells’ and another five ‘control wells’ were filled with 200 µL one-quarter Ringer’s solution. The *A. castellanii* plates were then incubated for 4, 6 or 8 h at 28 °C, and the *A. polyphaga* plates were incubated for 8 h. For *A. castellanii,* 50 µL of XTT menadione reagent (ATCC, Manassas, VA, USA); end concentration 0.3 mg XTT mL^−1^ and 100 µM menadione) was added to each well at each sampling time. For *A. polyphaga,* the CL solution in the ‘testing wells’ was aspirated after incubation, after which 200 µL Ringer’s solution was added and then 50 µL of the XTT menadione reagent was added. A difference in procedures was chosen because *A*. *polyphaga* needed a longer incubation time with XTT menadione to observe the metabolic activity made visible by XTT. The plates for *A. castellanii* were then incubated for 2 h at 37 °C and the plates for *A. polyphaga* for 24 h at 37 °C. Afterwards, the optical density (OD) values were measured at 450 nm with a reference filter of 620 nm (Genesys^TM^ 30, ThermoSpectronic, Avantor VWR, Pennsylvania, PA, USA). If a 100% reduction in the metabolic activity was measured in one of the ‘testing wells’, the content of this well was transferred to a nutrient agar (Oxoid Limited, Hampshire, UK) with *Enterobacter aerogenes* (ATCC 13048) overlay and incubated at 28 °C for two weeks, after which we examined the culture plates manually under a Leica DM IRB inverted microscope (200–400× magnification) for living trophozoites leaving a feeding trace in the bacterial layer.

### 2.4. Statistical Analyses

For the SK log reduction method, the CL solution’s effectiveness was expressed as the mean log reduction in the growth of trophozoites and cysts. One-way ANOVA and Dunnett’s test were used to compare the mean log reductions of the CL solutions with the control solution. For the XTT colorimetric assay, the CL solution’s effectiveness was expressed as a percentage of the OD reduction. The graph bars represent the means ± standard error of the means (SEM). The analyses were performed in GraphPad Prism (GraphPad Software, San Diego, CA, USA; version 6.0). A *p*-value ≤ 0.05 was considered statistically significant.

## 3. Results

### 3.1. Spearman–Karber Log Reduction Method: Trophozoites

**Figure 1:**
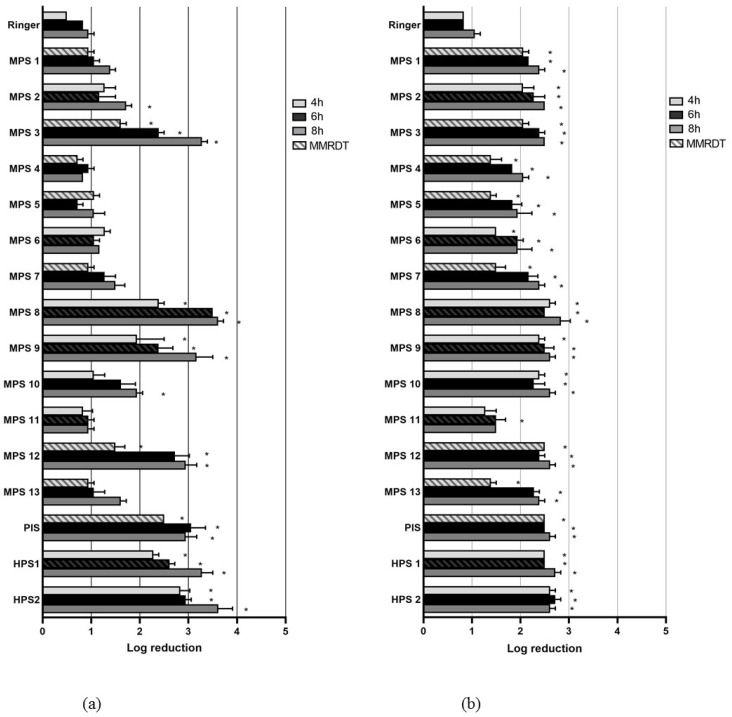
Mean log reduction of *Acanthamoeba* trophozoites after 4, 6 and 8 h incubation with one-quarter Ringer’s solution (control) or CL solution for *A. castellanii* (**a**) and *A. polyphaga* (**b**). * *p* = < 0.05.

Figure 1 shows the mean log reduction (MLR) of *Acanthamoeba* trophozoites for the control solution and the 16 CL solutions after 4, 6 and 8 h. For *A. castellanii,* ten MPS did not reach an MLR of three and were therefore considered ineffective. Three MPS, PIS and both HPS once exceeded an MLR of more than three at a minimum of one sampling time. At the MMRDT, however, only MPS 8 reached this value. For *A. polyphaga*, the MLR values varied less between the different CL solutions. At the MMRDT, all CL solutions had MLR values that differed statistically significantly from the control solution, but none of the solutions reached an MLR value of three.

### 3.2. Spearman–Karber Log Reduction Method: Cysts

**Figure 2:**
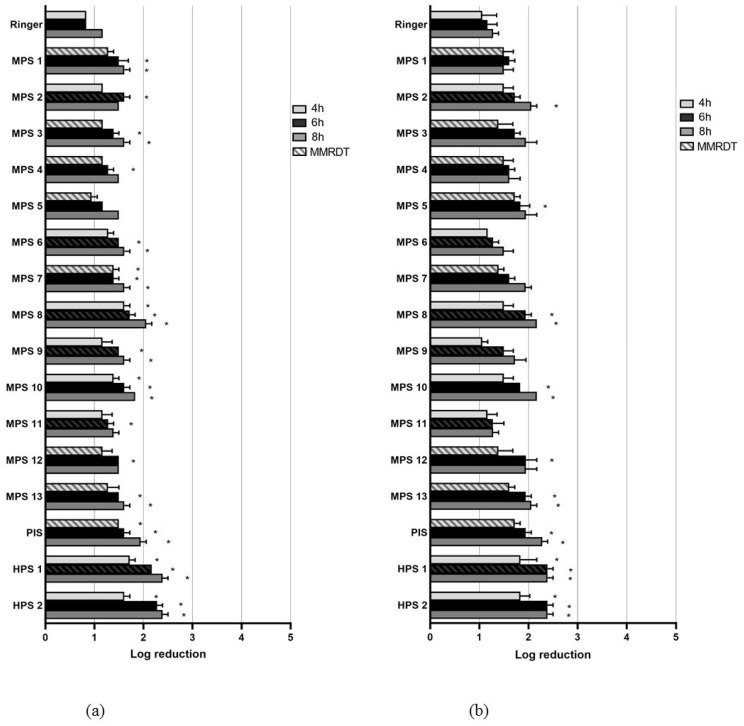
Mean log reduction of *Acanthamoeba* cysts after 4, 6 and 8 h incubation with one-quarter Ringer’s solution (control) or CL solution for *A. castellanii* (**a**) and *A. polyphaga* (**b**). * *p* = < 0.05.

In Figure 2, the MLR of *Acanthamoeba* cysts is presented for the control solution and the 16 CL solutions after 4, 6 and 8 h. None of the solutions reached an MLR of three. The MLR values for *A. castellanii* varied between 0.94 and 2.39 and for *A. polyphaga* between 1.06 and 2.39. At MMRDT, seven MPS, PIS and both HPS were statistically significantly more effective than the control solution for *A. castellanii.* For *A. polyphaga*, this was true only for MPS 8 and HPS 1.

### 3.3. XTT Colorimetric Assay and Residual Growth Analysis

**Figure 3:**
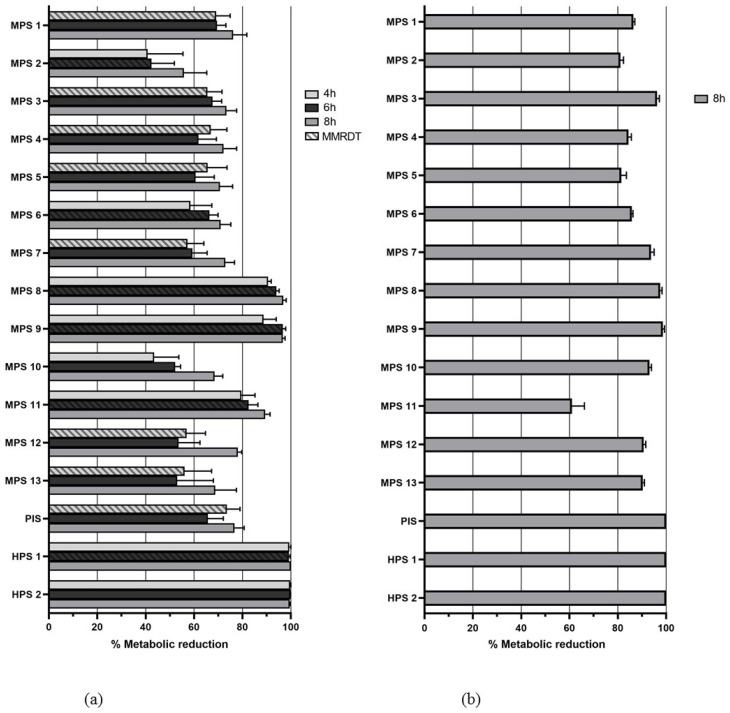
(**a**) Mean reduction in metabolic activity of *A. castellanii* trophozoites after 4, 6 and 8 h incubation per CL solution; (**b**) mean reduction in metabolic activity of *A. polyphaga* trophozoites after 8 h incubation per CL solution.

Figure 3 presents mean reductions in the metabolic activity of *A. castellanii* and *A. polyphaga*. At the MMRDT, none of the solutions reached 99.9% mean reduction (equivalent to 3 log reduction) against *A. castellanii.* HPS1 reached 100% mean reduction after 8 h. All solutions showed a better or at least equal reduction after 8 h, compared to incubation times of 4 and 6 h. For *A. polyphaga*, PIS and both HPS had a mean reduction of 100% after 8 h. Five solutions (MPS 8, MPS 9, PIS and both HPS) showed 100% reduction in one of the wells at a minimum of one time point against *A. castellanii* and/or *A. polyphaga*. When the content of these wells was transferred in triplicate to a nutrient agar with *E. coli* overlay, three out of three plates for the five solutions showed growth of trophozoites after two weeks for both *A. castellanii* and *A. polyphaga.*

## 4. Discussion

In this study describing the effectiveness of CL solutions available on the Dutch market against *Acanthamoeba* species, none of the tested CL solutions were consistently effective against trophozoites and cysts at the MMRDT. The results reflect the current lack of requirements for anti-amoebic activity of CL solutions in manufacturing protocols. Since *Acanthamoeba* keratitis in developed countries is almost universally associated with CL use and, therefore, a largely preventable disease, the effectiveness of CL solutions against *Acanthamoeba* species remains an important topic [4,5].

Both the classic SK log reduction method and an XTT colorimetric assay were used in this study; the latter being less time consuming and less subject to interobserver variation. A direct comparison of reduction numbers between the two methods is not possible, as the XTT colorimetric assay only detects the presence of metabolically active trophozoites. However, according to both methods, MPS 8 and 9 and both HPS were the most effective against trophozoites. MPS 8 and 9 contain a combination of active ingredients in contrast with the single PHMB systems that seem to be less effective, as was found by Fedorko et al. [9]. The efficacies of MPS 8 on trophozoites in other studies were comparably high: Kilvington et al. presented a 3.6 and 3.7 log reduction of, respectively, *A. castellanii* and *A. polyphaga* after 6 h [8]. MPS 9 yielded total kill (defined as >3 log reduction) of *A. castellanii* (ATCC 50373) trophozoites in a study by Üstüntürk [22]. The relatively high efficacies of HPS against *Acanthamoeba* trophozoites were also described by others [7,8,23]. Hydrogen peroxide is a very strong disinfectant, also against cysts, but loses part of its effectiveness in the neutralizing step that is required before contact with the cornea because of toxicity to the epithelium [8]. Kilvington et al. showed the cysticidal effects of HPS of 2.3 and 1.4 log reduction against *A. castellanii* and *A. polyphaga*, respectively, comparable to 1.5 to 2.5 log reductions by HPS 1 and HPS 2 in our study [8]. Only Kobayashi et al. found higher efficacies of HPS against one-week-old cysts of *A. castellanii* [7]. A different strain (ATCC 50514) was used in their study and the concentration of hydrogen peroxide was higher in one of their selected HPS, but this does not sufficiently explain the differences. The reproducibility of results from testing cysts in particular has been described to be difficult [9]. Part of the variability within test replications is probably caused by trophozoites and cysts sticking to plastic surfaces when preparing solutions and dilutions [24]. We therefore performed all experiments in triplicate.

This study is important as it is the first in its kind to describe the amoebicidal efficacy of the most used CL solutions present on the Dutch market. We took great care in selecting the most used combinations of active ingredients of CL solutions, and the number of tested solutions was large. To our knowledge, it is the first study combining the SK log reduction method and the XTT colorimetric assay for this purpose. Although the reduction values by the two methods are not directly comparable, the objective and efficient XTT colorimetric assay is potentially valuable for high-throughput settings [17]. Our results clearly confirm the relative ineffectiveness of single PHMB CL solutions compared to dual-biocide systems and PIS and HPS. A remarkable finding was a 0.5–1.3 reduction of trophozoites and cysts in the control one-quarter Ringer’s solution. In the study by Kobayashi et al., the same was found for one-week-old cysts [7]. As micro-organisms are starving in this medium, some reduction could be expected. Fedorko et al. suggested the use of Page’s amoeba saline and describe consistent results for trophozoites and cysts, but the results are not shown [9]. An attempt to use PYG medium resulted in granular precipitates in our hands and could therefore not be used. In addition to this, XTT colorimetric assays for *A. polyphaga* with four and six hour incubation times were logistically unfeasible. However, the eight hour incubation time, simulating overnight exposure to the CL solution, might be as interesting as the MMRDT. Since reduction values for most CL solutions were higher after longer incubation times, CL users might be advised to adhere to longer exposure times. In a recent case control study among 221 patients with AK and 1020 controls, 47% said to keep their CLs immersed in CL solution for at least eight hours [25]. This is a possible explanation for the discrepancy between incidence numbers and the worrisome results of the current study. Other authors also found that reduction values increased at increasing CL solution exposure times, up to 24 h [7,12]. Lastly, it should be noted that the selected CL solutions were all intended for soft CLs, while 18% of AK patients reported wearing rigid-gas permeable CL in a Dutch national survey [5].

In conclusion, soft CL solutions present on the Dutch market today do, in general, show insufficient amoebicidal efficacy against *Acanthamoeba* trophozoites and cysts. This poses a risk for AK in CL users. We affirm the previously posed recommendation that CL solution manufacturing protocols include testing of anti-amoebic effectiveness not only for soft CL solutions but also for solutions used in rigid-gas permeable CL wear [9,14]. The development of a standard protocol, as is expected to be released soon by the International Organization for Standardization, will aid in comparing different CL solutions on the market and make scientific studies on this subject comparable [10]. In the long run, this will improve the anti-amoebic effectiveness of CL solutions. In the meantime, strict hygiene practices should be emphasized to CL users, as CL solutions do not yet provide the desired barrier against *Acanthamoeba* species.

## Data Availability

The dataset used during the current study is available from the corresponding author upon reasonable request.

## Supplementary Materials

The following supporting information can be downloaded at: https://www.mdpi.com/article/10.3390/pathogens12020214/s1, Table S1: Selected contact lens solutions and their ingredients.
