# Supplementary material for: In Vitro Effectiveness of Soft Contact Lens Solutions Available on the Dutch Market against Acanthamoeba Species
Source: Pathogens. 2023 Jan 29;12(2):214. doi: 10.3390/pathogens12020214 (PMC9959425; doi:10.3390/pathogens12020214)
Supplement: Supplementary file 1 [file pathogens-12-00214-s001.zip › pathogens-2133108-supplementary.pdf]

Table S1. Selected contact lens solutions and their ingredients.

Code	Product name	Manufacturer	Active ingredient (% w/v)	Surfactant	Buffer	EDTA	Other	MMRDT (hours)
MPS 1	Albert Heijn All-in-one	Melleson Pharma BV	PHMB 0.0001			0.01%	Isotonic increasing ingredients	4
MPS 2	HEMA no-rub solution for monthly soft lenses	Ridam Care	PHMB 0.0002	Pluronic	Borax, boric acid	0.01%		6
MPS 3	Kruidvat Opticare Zachte lenzen All-in-One	I-care Vision BV	PHMB 0.0004	Poloxamer 407	Borax, boric acid	0.01%		4
MPS 4	Eyewish Eyexpert Easycare	Cooper Vision	PHMB 0.0001	Poloxamer 188	Phosphate	0.01%		4
MPS 5	Specsavers easyvision easypurpose multipurpose solution	Cooper Vision	PHMB 0.00013	Poloxamer	Phosphate	% n.a.	Hyaluronate 0.01%	4
MPS 6	Ote Sensation All-in-one	Oté Pharma Sol BV	PHMB, polyquaternium				Hyaluronate	6
MPS 7	ClearVision	Oté Pharma Sol BV	PHMB 0.0001	Poloxamine 1%			Hyaluronate, provitamin B5	4
MPS 8	Acuvue Revitalens	Johnson & Johnson	Polyquaternium 0.0003, Alexidine 0.00016	Tetronic 904	Borax, boric acid, trisodium citrate dihydrate	% n.a.	Sodium chloride	6
MPS 9	OptiFree PureMoist	Alcon Laboratories	Polyquad 0.001, Aldox 0.0006	Tetronic 1304 (poloxamine)	Boric acid, sodium citrate	0.01%	Hydra-Glyde (EOBO-41), sodium chloride	6
MPS 10	Solocare Aqua	Menicon	Polyhexanide 0.0001	Poloxamer 407	Sodium phosphate	% n.a.	Sorbitol, provitamin B5, tromethamin, lens case with silver ions	5 minutes
MPS 11	Regard	Vita Research	Chlorite, hydrogen peroxide 0.01	Poloxamine, Pluronic F-68	Boric acid		HPMC 0.15%	6
MPS 12	Biotrue	Bausch & Lomb	PAPB 0.00013, Polyquad 0.0001	Poloxamine	Boric acid, sodium borate	% n.a.	Hyaluronate, sulfobetaine, sodium chloride	4
MPS 13	Pearl iWear Multibalance All-in-1	Avizor S.A.	PHMB 0.0002	Poloxamer		0.01%	Allantoine, hyaluronate	4
PIS	Cleadew	Ophtecs Corporation	Povidone iodine 4.0mg/tablet, H202 40 ppm		Boric acid, ascorbic acid	% n.a.		4
HPS 1	Easysept	Bausch & Lomb	H2O2 3.0		Phosphoric acid, phosphate		Sodium chloride	6
HPS 2	Avizor Everclean	Avizor S.A.	H2O2 3.0					2

* Abbreviations: EDTA = ethylenediaminetetraacetic acid, H2O2 = hydrogen peroxide, HPMC = hydroxypropylmethylcellulose, HPS = hydrogen peroxide solution, MMRDT = manufacturer's minimum recommended disinfection time, MPS = multipurpose solution, PAPB = polyaminopropyl biguanide, PHMB = polyhexamethylene biguanide, PIS = povidone iodine solution, n.a. = not available.
